# Gray Bananas and a Red Letter A — From Synesthetic Sensation to Memory Colors

**DOI:** 10.1177/2041669518777515

**Published:** 2018-05-31

**Authors:** Franziska Weiss, Mark W. Greenlee, Gregor Volberg

**Affiliations:** University of Regensburg, Germany

**Keywords:** color, multisensory processing, perception, synesthesia, cross-activation theory, conceptual-mediation model, memory colors

## Abstract

Grapheme–color synesthesia is a condition in which objectively achromatic graphemes induce concurrent color experiences. While it was long thought that the colors emerge during perception, there is growing support for the view that colors are integral to synesthetes’ cognitive representations of graphemes. In this work, we review evidence for two opposing theories positing either a perceptual or cognitive origin of concurrent colors: the cross-activation theory and the conceptual-mediation model. The review covers results on inducer and concurrent color processing as well as findings concerning the brain structure and grapheme–color mappings in synesthetes and trained mappings in nonsynesthetes. The results support different aspects of both theories. Finally, we discuss how research on memory colors could provide a new perspective in the debate about the level of processing at which the synesthetic colors occur.

## Introduction

Synesthesia is a phenomenon in which a stimulus (inducer) elicits an additional, unstimulated experience (concurrent; Grossenbacher & Lovelace, 2001). One of the most common forms is grapheme–color synesthesia in which the experience of a grapheme is accompanied by the experience of color. These color concurrents arise automatically and cannot be voluntarily controlled (for an overview, see Mattingley, 2009; Rich, Bradshaw, & Mattingley, 2005). The inducer–concurrent combinations vary across individuals but remain stable over time (Ward, 2013; cf. Simner, Ipser, Smees, & Alvarez, 2017).

Current prevalence rates for synesthesia range from 0.08% estimated from responses to a newspaper article on synesthesia (Rich et al., 2005), to 4.4% based on experimental data in large-scale samples (Simner et al., 2006). For the grapheme–color subtype, the results of recent studies broadly converge on a prevalence rate around 1% (Carmichael, Down, Shillcock, Eagleman, & Simner, 2015; Watson et al., 2017). Since a large proportion of synesthesia research is about the grapheme–color experience, our review focuses on this subtype.

Synesthesia can be diagnosed with different established tests. A color-picker task (e.g., Eagleman, Kagan, Nelson, Sagaram, & Sarma, 2007) determines the variability of the color matches selected for specific graphemes in multiple randomized trials. The resulting color variation score is the average Euclidean distance of the color coordinates in RGB color space. Synesthetes demonstrate a characteristically low variability and achieve low color variation scores. Nonsynesthetes, on the other hand, have difficulties to select the same color for a given grapheme across trials and produce high variation scores. The outcome of the color picker test can be validated in a speeded congruency task (Eagleman et al., 2007) in which participants need to quickly decide whether the print color of a grapheme is congruent or incongruent to the concurrent. Synesthetes typically respond faster and more accurately than nonsynesthetes. Finally, questionnaires can be used to assess the quality of the color experience in synesthetes (Barnett & Newell, 2008; Hossain, Simner, & Ipser, 2017). However, as of today, Eagleman’s (2007) synesthesia battery seems to be the most widespread and the only validated diagnostic test (Carmichael et al., 2015).

The current knowledge on synesthetic experiences does not only rely on subjective reports but also on experiments demonstrating a behavioral effect of synesthetic colors. For instance, multiple studies show a synesthetic Stroop effect. In the synesthetic Stroop task (Wollen & Ruggerio, 1983), the inducers are printed in a color congruent, incongruent, or neutral with respect to the induced color. The pattern of results is similar to that of a classical Stroop task (naming the print color of a color word, e.g., the word ‘blue’ printed in red; MacLeod, 1991) with higher reaction times on incongruent trials due to a response conflict (Dixon, Smilek, & Merikle, 2004; Nikolić, Lichti, & Singer, 2007; Ward, Li, Salih, & Sagiv, 2007).

Despite the evidence of synesthesia being a genuine phenomenon, it is still unclear how inducers and concurrents are processed to generate this experience. Here, we review findings in regard to two prominent and well-elaborated models with opposing positions in current synesthesia research and propose a future research perspective.

## Two Current Theories on Synesthesia

Grapheme–color synesthesia is an example of involuntary nonretinal vision (Pearson & Westbrook, 2015): The color experience does not correspond to the spectral properties of the inducer and associated activity in wavelength-tuned photoreceptors. Thus, a central concern in synesthesia research is how the color experience emerges. Different authors would place the critical event on different levels of the processing hierarchy from sensory registration to object recognition and related semantic operations. Accordingly, models of synesthesia have been characterized based on the time course of activation (early or late; Brang, Hubbard, Coulson, Huang, & Ramachandran, 2010; Volberg, Karmann, Birkner, & Greenlee, 2013) or by the processing level (low or high; Janik McErlean & Banissy, 2017; Mroczko-Wąsowicz & Nikolić, 2014).

In this section, we will focus on two models that could be considered as two ends of the continuum from low- to high-level explanations of the origin of synesthetic colors: the cross-activation theory (Hubbard, Brang, & Ramachandran, 2011) and the conceptual-mediation model (Chiou & Rich, 2014). The former model posits that synesthesia arises during sensory processing due to an irregular activation of neurons encoding the concurrent feature. The latter model suggests that colors are part of the object knowledge that is linked to the conceptual representation of the grapheme. Synesthetic colors would then arise through memory retrieval not perceptual processes.

One major model that will not be examined in detail is the disinhibited-feedback model (Grossenbacher & Lovelace, 2001). It resembles the cross-activation theory in that synesthetic color experiences involve irregular activity in color coding neurons but assumes a different propagation of neural activation from the inducer to the concurrent pathway. Thus, it can be regarded as a similar position to that of the cross-activation theory.

### Cross-Activation Theory

The cross-activation theory is a low-level model that specifically addresses grapheme–color synesthesia. Accordingly, the focus is on brain areas that are associated with the feature analysis of graphemes and colors (Hubbard et al., 2011; Ramachandran & Hubbard, 2001). The pertinent brain site for grapheme processing is the visual word form area (VWFA; McCandliss, Cohen, & Dehaene, 2003; see Grotheer, Ambrus, & Kovács, 2016 for an analogous number form area). The VWFA is known to be active during reading and is thought to support the translation of physical grapheme shapes into letter and word meaning. Adjacent to the VWFA in the posterior occipito-temporal cortex lies the visual area 4 (V4). The majority of V4 neurons are color selective (Roe et al., 2012), and lesions in that area produce a loss of color vision in humans (Bartolomeo, Bachoud-Lévi, & Thiebaut de Schotten, 2014). Thus, V4 can be characterized as a color-processing area.

According to the cross-activation theory, synesthesia is a neurological condition with increased neural cross-wiring between V4 and VWFA. As a consequence, a neural representation of a grapheme in VWFA would automatically entail a representation of color in V4 and generate the percept of a colored grapheme. Thus, proponents of the cross-activation theory believe that synesthetic experiences emerge from a unique neuroanatomical characteristic of the synesthetes’ brains. Moreover, they assume that synesthesia emerges at the earliest representational levels of grapheme and color processing.

Since the original publication, two modifications have been made on the cross-activation theory. First, it was acknowledged that distributed representations of grapheme shape and color must be integrated in order to generate a unified percept of a colored grapheme (Hubbard et al., 2011). As in normal vision, the integration would involve activity in parietal cortex that has a common spatial reference map for unisensory feature representations (Robertson, 2003). Second, in response to new developments in the research of grapheme processing, letter recognition is no longer seen as a result of template matching but as an incremental analysis of the features that make up the grapheme. Thus, the color inducing entity might not be the grapheme itself but rather a feature thereof (cascaded cross-tuning model; Brang et al., 2010; Hubbard et al., 2011). In sum, in the revised version of the cross-activation theory grapheme processing relies on feature analysis, and the conscious perception of the colored grapheme relies on feature binding. However, early stage cross-activation is still stated as the essential first step of atypical color experiences in synesthetes (Hubbard et al., 2011).

### Conceptual-Mediation Model

Contrary to low-level explanations of synesthetic experiences, high-level conceptual theories focus on the associative aspects of inducers as triggering factors for the concurrent. These theories are unspecific with respect to the inducer or concurrent modalities, and can thus explain perceptual inducers that rely on sensory events, for example, seeing written graphemes or hearing sounds, as well as conceptual inducers that do not (Mroczko-Wąsowicz & Nikolić, 2014).

The conceptual-mediation model of synesthesia by Chiou and Rich (2014) is based on a model of object knowledge (Lambon Ralph & Patterson, 2008; Patterson, Nestor, & Rogers, 2007). It proposes that the color activation in grapheme–color synesthesia relies on cognitive rather than sensory representations of the inducer and the concurrent. Accordingly, both synesthetes and nonsynesthetes would process a grapheme alike until a conceptual representation is activated. While in both groups, the grapheme representation is the center of a semantic network (“hub”) with many attributes (“spokes,” e.g., lexical and phonetic information for graphemes), synesthetes also have a spoke for the typical color of this grapheme. Thus, synesthetic colors are stored within a semantic network as memory colors for grapheme objects (Chiou & Rich, 2014).

Based on previous work on object knowledge representation, Chiou and Rich (2014) suggest that the anterior temporal lobe (ATL) is the central hub that integrates the concept of the grapheme. When the grapheme concept is accessed, its semantic network is activated in the ATL. Depending on the actual spokes, the ATL might activate a concurrent color representation in V4 but also in higher brain areas where color knowledge is represented. The spread of activation within the concurrent processing network would then affect the strength of the synesthetic experience (i.e., *seeing* or *feeling* the synesthetic color). Damage or disruption of ATL function would impede the color coactivation and erase the synesthetic experience.

## Theoretical Predictions

To interpret experimental results for or against the existing theories, it is important to clarify what these theories predict. We organize the predictions, as well as the review of empirical results, as follows. The “Predictions Regarding Inducers” section focuses on inducers and the conditions under which graphemes produce color experiences in synesthetes. In the next section, we turn our attention to the concurrent, and whether synesthetic color experiences are similar to real color sensations. In the following section, we discuss differences between synesthetes and nonsynesthetes in neural structure and function specifically predicted by one of the reviewed theories. Finally, we address the question of whether there are canonical mappings between graphemes and colors. In doing so, we proceed from cognitive or perceptual functions to synesthetic individuals and to synesthetic mappings across individuals while concentrating on the two competing theories on synesthesia. In this respect, we offer a different perspective from that of recent review that mainly focused on color processing in synesthetes (Janik McErlean & Banissy, 2017).

### Predictions Regarding Inducers

Graphemes are a special class of objects with distinct visual features like lines, curves, angles, and proportions. Simultaneously, as lexical symbols, they also contain abstract information, for example, the magnitude of numerals or the use of letters in language. Hence, we differentiate between the early *perceptual* representation of visual features and the late *conceptual* representation of abstract information about the identified graphemic object.

If synesthetic colors are directly coactivated through neural connections with grapheme-processing areas, then the synesthetic experience should depend on low-level physical properties of the inducer, for example, angle or global shape (possibly depending on font and size) of a letter, and presentation modality, for example, written versus spoken. Furthermore, the inducer must be physically present so that its features can be processed on a perceptual level. If features of graphemes lead to synesthetic experiences, this should also apply to letter-like objects (fake letters) with similar features. Conscious recognition of a grapheme, on the other hand, should not be essential for the concurrent to arise. Accordingly, manipulations of attention modulating conscious access to the inducer should have little impact on the synesthetic experience.

In contrast, according to high-level theories like the conceptual-mediation model, the physical form of the inducer should not be critical for the concurrent color sensation. An inducer does not even need to be physically present if its concept is activated. The synesthetic experience depends on the conceptual processing of the inducer, including its conscious identification (prior to the synesthetic experience). Since attention is important to consciously perceive a stimulus, it is likely to modulate the experience of a concurrent color.

### Predictions Regarding Concurrent Colors

Proponents of the cross-activation theory view the synesthetic experience as a perceptual phenomenon. If the color is coactivated during bottom-up processing, then synesthetic colors should be similar to real colors. That is, achromatic inducers should produce the same experiences and behavioral effects in synesthetes as chromatic graphemes do in nonsynesthetes. Further, color-processing areas like V4 should be active in response to achromatic inducers, and this activity should occur early during inducer processing, almost simultaneously to activity in the VWFA.

The conceptual-mediation model does not allow strict predictions about the nature of concurrent colors. Similarly to memory colors, induced colors might be represented within a conceptual network, or perceptually with corresponding activations in V4, depending on the strength of synesthetic associations. The main difference is the route of activation of the concurrent processing, which is fast and bottom-up in the cross-activation theory and delayed and top-down in the conceptual-mediation model.

### Predictions Regarding the Brain Structure and Function

The most prominent prediction that can be derived from the cross-activation theory is a structural hyperconnectivity in the brain of synesthetes, specifically between the inducer-processing and the concurrent-processing brain areas. That is, in grapheme–color synesthesia, the letter-sensitive VWFA and the color-processing V4 should exhibit structural differences between synesthetes and neurotypical individuals, especially enhanced fiber tracks between those areas.

The conceptual-mediation model does not presuppose any structural brain differences between synesthetes and nonsynesthetes. Widespread functional activity related to memory retrieval is plausible in areas including associative visual cortex and the ATL. Such findings, however, would not contradict the predictions from the cross-activation theory.

### Predictions Regarding Inducer–Concurrent Mappings

According to the cross-activation theory, color coactivation relies on grapheme features, that is, straight or curved lines with a given orientation. There is no reason to assume that these features, or combinations thereof, consistently map to a specific color across synesthetes.

Conversely, the conceptual-mediation model states that inducer and concurrent are linked in an object knowledge network. It is evident that these links must be acquired before any synesthetic color experience can occur. Because children acquire literacy in a common environment within a given culture, with similar toys and educational materials, it is fair to assume that some grapheme–color mappings should be highly prevalent across the population of synesthetes. Another implication is that, after excessive training with precolored graphemes, also nonsynesthetes should experience concurrent colors with achromatic letters and numbers.

## Empirical Findings

### Findings Regarding the Inducer

#### Inducer features

Since graphemes are characterized by distinct visual features, it is plausible to assume that grapheme–color synesthesia emerges during sensory processing of the visual input (Brang et al., 2010). If this was the case, then changing the features of a grapheme should also change the concurrently perceived color.

Indeed, case reports show that the font type and capitalization of the inducer can affect the hue or the vividness of the concurrent color (Ramachandran & Hubbard, 2001) and that synesthetic colors can transfer to perceptually similar graphemes of a previously unknown alphabet in synesthetes (Mroczko, Metzinger, Singer, & Nikolić, 2009). Furthermore, it has been reported in a large-scale study that similarly shaped graphemes induce similar concurrent hues in individual synesthetes (Brang, Rouw, Ramachandran, & Coulson, 2011). The authors obtained color picks for a full set of graphemes and computed the color distance for each possible pairing. The data were then correlated with measures describing the perceived similarity between the relevant letters, as revealed by a different study (Courrieu, Farioli, & Grainger, 2004). These measures did not reflect the perceived inducer similarity in the sample of synesthetes and did not rely on physical features of the graphemes, like orientation or curvature. Thus, the data do not show a direct link between physical inducer features and concurrent colors.

In a related study, Ramachandran and Marcus (2017) recently investigated form and color binding for nongrapheme stimuli in synesthetes. After adaptation to alternating vertical and horizontal stripes drawn in opponent colors, observers typically show an orientation-contingent color aftereffect, the so-called McCollough effect. The McCollough effect was found to persist longer in synesthetes compared to controls (see also Blake, Palmeri, Marois, & Kim, 2005). This finding points to the existence of stronger form and color connections in this group. However the authors do not exclude that the effect was driven by back projections from higher visual processing areas (Ramachandran & Marcus, 2017).

It should be noted that the reported concordances between grapheme shape and concurrent color would not exclusively support a low-level explanation of synesthesia. It is also possible that in the course of literacy acquisition, new graphemes are compared to those that have already been learned and subsumed under the corresponding color association before their own color variant is established. Similarly shaped graphemes might be associated with a similar color until subtle differences in these graphemes are learned leading to stronger hue differentiation. Indeed, developmental studies with synesthetes show that inducer–concurrent pairings emerge progressively rather than instantaneously (Cohen Kadosh & Terhune, 2012; Simner & Bain, 2013; Simner, Harrold, Creed, Monro, & Foulkes, 2009).

#### Inducer meaning

Most synesthetes report experiencing their concurrents regardless of the presentation modality of the inducer (e.g., visual vs. auditory, Arnold, Wegener, Brown, & Mattingley, 2012; Rich et al., 2005). In line with these findings, a number of studies have revealed that not the physical form of the inducer but rather the semantic representation of that grapheme is critical for the concurrent color activation (Chiou & Rich, 2014; Grossenbacher & Lovelace, 2001; Rich et al., 2005). For example, it was found that ambiguous graphemes induce different colors depending on the context in which they are presented. For example, the grapheme ‘5’ is typically perceived as the letter ‘S’ or the digit ‘five’ when presented within flanking letters or numbers, respectively. The induced colors are different in these situations although the physical form of the inducer is identical (Dixon, Smilek, Duffy, Zanna, & Merikle, 2006).

Comparable results were obtained in a letter rotation task with graphemes that change identity depending on their orientation (e.g., 6–9). Synesthetes reported changes of synesthetic colors as the orientation of graphemes changed to an ambiguous form. Similarly, graphemes morphing into each other induced changes in the synesthetic color corresponding to the respective dominating letter percept (Bridgeman, Winter, & Tseng, 2010).

While Bridgeman et al. (2010) gathered subjective reports on the experienced color, Kim, Blake, & Kim (2013) required a speeded categorization of either the synesthetic color or the grapheme identity of these types of ambiguous characters (e.g., 6/9, M/W). An achromatic grapheme was presented in one of the seven different orientations, such that it would be unequivocally identifiable at 0° and 180° (e.g., 6 or 9) and ambiguous at 90°. The results showed that the reaction speed not only for grapheme but also color identification parametrically varied with the stimulus orientation, with the highest reaction times for the maximum angular deviation from an upright grapheme presentation.

If concurrent colors are bound to semantic grapheme representations, then physically different graphemes should activate the same color if they are mapped to the same semantic representation. This idea was tested in a semantic learning task in which synesthetes learned novel graphemes from an ancient writing system with a one-to-one mapping to the letters of the Latin alphabet (Mroczko et al., 2009). In line with the predictions, different graphemes with the same lexical meaning activated the same color concurrents. Importantly, synesthetes developed these new associations within minutes, which cannot be explained by mere low-level perceptual learning that would only develop over much longer time intervals (Jürgens & Nikolić, 2012).

Overall, the available studies show that the semantic or spatial context in which a grapheme is presented modulates the induced color for synesthetes and that different graphemes with the same meaning can map to the same color. This supports the idea that synesthetic colors are bound to high-level, nonperceptual representations of the grapheme. It should be noted that Dixon et al. (2006) reported single case data, with unclear relevance at the population level. The evaluation of the study results where rotated or morphed graphemes were used is not straightforward either (Bridgeman et al., 2010; Kim et al., 2013): Morphing and rotating the grapheme do not only affect high-level grapheme representations but also low-level feature representations. Thus, the results are also compatible with the view that synesthesia relies on cross-activation during the perceptual processing of the grapheme. Nevertheless, the finding that different graphemes evoke the same color if they have the same meaning is a convincing argument that semantic representations are crucial for synesthesia, in line with a conceptual-mediation approach.

#### Conceptual inducers

The results of the studies reviewed in the previous sections suggest that grapheme features activate semantic grapheme representations, which then coactivate colors. Interestingly, a physical stimulus and sensory processing do not even seem necessary to induce color in some number–color synesthetes (Dixon, Smilek, Cudahy, & Merikle, 2000; Jansari, Spiller, & Redfern, 2006).

Dixon et al. (2000) investigated a single case of a digit–color synesthete that showed high consistency on multiple color namings for each digit (1–9). In a synesthetic Stroop task with color patches (baseline), and digits colored in their synesthetic color (congruent) or a different color (incongruent), the synesthete showed significantly slower reaction times naming the print color on incongruent trials than on baseline or congruent trials. In an arithmetic variation of the Stroop task, an arithmetic operation was followed by a color patch congruent or incongruent to the synesthetic color for the calculation’s solution. Again, the synesthete showed slower reaction times naming the patch color on incongruent trials. None of these effects was found in any of the eight nonsynesthetic control participants (Dixon et al., 2000).

It should also be noted that there are some other subtypes of synesthesia in which the inducer is a concept and not a physical stimulus. One such type is swimming-style–color synesthesia (Nikolić, Jürgens, Rothen, Meier, & Mroczko, 2011; Rothen, Nikolić, et al., 2013), where color concurrents can be elicited by illustrations of swim strokes. Another variant is the very common time unit synesthesia (e.g., days or months associated with color). Because time units are not physical entities, and there is no sensory organ for time perception, it must be the concept of the time unit that acts as the inducer (Mroczko-Wąsowicz & Nikolić, 2014). Thus, synesthetic experiences do not appear to depend on the sensory processing of the inducer, at least in some forms of synesthesia.

#### Inducer recognition

Object recognition depends on how the outcome of sensory processing is selected for further action or cognition, that is, how attention is allocated to the outcome (Robertson, 2003). If semantic grapheme representations are crucial in synesthesia, then concurrent colors should emerge only when conscious access to a conceptual representation of the grapheme is possible. When conscious access is limited, for example, by masking of the grapheme or by detracting attention from the task, then no color experience should occur.

This prediction was tested using a Stroop task with synesthetes and a control sample (Mattingley, Rich, Yelland, & Bradshaw, 2001; see also Rich & Mattingley, 2003, 2010; Rich et al., 2006; Sagiv, Heer, & Robertson, 2006). Both groups showed the expected interference effect in a standard Stroop task. In a synesthetic Stroop with synesthetically congruent or incongruent graphemes as well as neutral symbols, only synesthetes showed a significant congruency effect. In another task variant, an achromatic grapheme prime was followed by a color patch which should be identified. The color of that patch was congruent, incongruent, or neutral relative to the synesthetic color of the prime stimulus. The prime was presented for either 500 ms, 56 ms, or 28 ms in separate experiments. The conscious awareness of the prime stimulus was tested by an additional condition with a grapheme identification task. Synesthetes only showed the typical Stroop interference when the prime was consciously available (500 ms). The results suggest that the synesthetic experience is contingent on conscious access to the inducer representation (Mattingley et al., 2001). Later experiments confirmed that finding and showed smaller congruency effects under high-attentional load conditions during divided attention (Mattingley, Payne, & Rich, 2006; Mattingley et al., 2001), if attention was distracted from an inducer during eye movements (Nijboer & der Stigchel, 2009) or if the allocation of spatial attention to the inducer was disrupted by transcranial magnetic stimulation (TMS) to the parietal cortex (Esterman, Verstynen, Ivry, & Robertson, 2006; Muggleton, Tsakanikos, Walsh, & Ward, 2007).

The role of attention was also shown with hierarchical stimuli where a global character is made up of multiple smaller local characters (Navon, 1977). Depending on the attended global or local level, synesthetes experience the synesthetic color of the grapheme corresponding to that level (Palmeri, Blake, Marois, Flanery, & Whetsell, 2002). Utilizing hierarchical stimuli, a Stroop-like color interference task has been carried out by Rich and Mattingley (2003). The local and global graphemes were either consistent (e.g., large A composed of smaller letter As) or inconsistent (e.g., large A composed of small Bs), and graphemes on the global and local levels were colored either congruently or incongruently relative to their synesthetic color. Synesthetes’ reaction times were higher for incongruent compared to congruent print and induced colors on one of the levels and were even higher if the print colors on both levels were incongruent relative to the induced color. Nonsynesthetes did not show any congruency effect (Rich & Mattingley, 2003). In a further experiment, participants were instructed to selectively attend to one level. The congruency effect was reduced for the unattended level, suggesting that lack of attention reduces the effects of synesthetic experience. However, the congruency effect was not totally absent. The authors argue that, similarly to attentional effects in the classic Stroop task, semantic representations of the synesthetic colors were activated less efficiently by the inducers (Rich & Mattingley, 2003). The changing color experience with hierarchical graphemes is consistent with the findings using ambiguous letters and only conceptually triggered inducers described earlier, in that the conceptual interpretation of the inducer is what elicits the concurrent, not the physical presentation.

### Findings Regarding the Concurrent Color

#### Synesthetic colors

If inducers directly coactivate color-sensitive neurons at an early processing stage, then synesthetic colors should produce quantifiable effects similar to real colors. Real–colored target stimuli can easily be detected within large sets of achromatic distractor stimuli in visual search tasks (Treisman & Gelade, 1980). While it is often argued that this color pop-out effect in visual search is also found in synesthetes (Palmeri et al., 2002; Ramachandran & Hubbard, 2001), a closer look at the data shows a difference between the benefit of synesthesia in these search tasks and pop out (see also Chiou & Rich, 2014). Synesthetes show higher accuracy in target detection than controls but do not show the lower reaction times for pop out compared to non-pop out displays that are typically found in visual search tasks with normal subjects (Rich & Karstoft, 2013; Wolfe, 1998).

Some basic phenomena in color vision that occur with real–colored stimuli can also be produced with induced colors, like the Stroop effect described earlier. However, it is not clear at which processing stage the response conflict of the Stroop effect arises. In a manipulation of the synesthetic Stroop task, synesthetes showed an opponent color effect (Nikolić et al., 2007), in that the response conflict on incongruent trials was higher when the print color and synesthetic color were opponent (e.g., red and green) compared to a reduced conflict for colors of different color channels (e.g., red and blue). The authors concluded that the synesthetic Stroop effect is based on early visual processing, especially since this opponency effect was not found in a semantic control task. This conclusion is at odds with studies reviewed in the previous section which suggested that response conflicts between induced and real colors rely on higher level representations of the inducer. However, it cannot be excluded that color-opponent neurons were activated top-down, subsequent to a high-level representation of the inducer and its color. Also, one might challenge the interpretation of the semantic control task presented by Nikolić et al. (2007). As control stimuli, they used a lemon (associated with yellow), a heart (associated with red), and a smiley (no color information given). While the lemon has been shown to be a color diagnostic object in studies of memory colors (Hansen, Olkkonen, Walter, & Gegenfurtner, 2006), the color associations for the two other shapes seem to be more vague. Both synesthetes and nonsynesthetes showed overall shorter reaction times for naming the color of the semantic stimuli than for graphemes, suggesting that the congruency effect for semantic stimuli was less due to response conflict on incongruent trials but rather due to facilitation on the congruent trials. Further, the observation of overall higher reaction times in the grapheme experiment compared to the semantic experiment precludes the possibility that responses in the former condition rely on initial color representations. It is plausible that the conflict arises at a higher level semantic processing stage for the graphemes as well and is then passed down to the color-opponent visual processing areas.

A separate line of investigation addresses whether the perceived color of a grapheme depends on the sensory and temporal context of the presentation, as is the case for real colors. For example, the sudden offset of a chromatic stimulus after prolonged viewing would produce an afterimage in the opponent color due to previous adaptation of color-coding neurons. Synesthetic color experiences do not change to opponent colors at grapheme offsets though (Bridgeman et al., 2010). Similarly, the synesthetic color experience does not depend on the brightness of the surround, as is the case with real colors (Erskine, Mattingley, & Arnold, 2013; Hong & Blake, 2008). Thus, induced color experiences are dissociable from regular color perception in synesthetes.

#### V4 activation

Functional magnetic resonance imaging (fMRI) has revealed that a variety of brain sites are involved in synesthesia, including temporal association areas and parietal areas (Laeng, Hugdahl, & Specht, 2011; Paulesu et al., 1995; Rich et al., 2006; Rouw, Scholte, & Colizoli, 2011; Weiss, Zilles, & Fink, 2005). The role of color-processing area V4 in synesthesia is still debated though. While some studies revealed higher blood oxygen level-dependent (BOLD) activation in V4 in response to inducers for synesthetes than controls (e.g., Hubbard & Ramachandran, 2005; Nunn et al., 2002; Sperling, Prvulovic, Linden, Singer, & Stirn, 2006), other studies failed to find V4 activation in synesthesia (Hupé, Bordier, & Dojat, 2012; Rich et al., 2006; Rouw & Scholte, 2007; Rouw et al., 2011; Sinke et al., 2012). Among the latter studies, two utilized demanding spatial localization or memory tasks (Hupé et al., 2012; Rich et al., 2006). Rouw and Scholte (2007) contrasted BOLD responses to both weak and strong inducers with those of noninducers, which possibly reduced the power of the analysis. Finally, Sinke et al. (2012) collapsed colored and uncolored versions of letters and nonletters in their analysis, so that V4 activation differences between the conditions were unlikely. Hupé and Dojat (2015) provide an in-depth review of neuroimaging studies on synesthesia. Two problems they identified are the use of different study designs and varying localization methods for V4. Another problem, addressed by Gould van Praag, Garfinkel, Ward, Bor, and Seth (2016), is the heterogeneity of synesthetic phenomena. They measured the neuronal activity in response to silent letter identification for inducing letters and noninducing symbols in 20 synesthetes and matched controls. While no significant group differences in activation were found in color-selective areas, BOLD responses correlated positively with scores in *localization* and *automaticity* factors of the Colored Letters and Numbers questionnaire (Rothen, Tsakanikos, Meier, & Ward, 2013). Synesthetes who experience their concurrent at a specific location showed stronger activity in response to inducing stimuli in their individual color-selective areas on both hemispheres. If the concurrent experience arises more automatically, synesthetes show higher activity in their left hemispheric color areas (Gould van Praag et al., 2016). This finding is in line with the assumption of a different activation extent within the color network dependent on the strength of the synesthetic association made by the conceptual-mediation model.

#### Time course of color coactivation

The time course of color coactivation gives further information on the role of low- and high-level mechanisms in grapheme–color synesthesia. Electro- and magnetoencephalography (EEG/MEG) provide an appropriate temporal resolution for that purpose.

Brang, Edwards, Ramachandran, & Coulson (2008) and Brang, Kanai, Ramachandran, & Coulson (2011) measured event-related potentials (ERPs) in synesthetes and controls while presenting color priming sentences that ended with a color word, a color inducing grapheme, or a color patch. These could be either congruent or incongruent to the context of the sentence. ERP responses for color terms and color patches were similar in both groups with a stronger N400 component on incongruent trials. The same effect was found for inducers in synesthetes but not in controls. More interestingly in the current context, the N1/P2 complex of the ERP showed a negative shift for color congruent compared to incongruent inducers in synesthetes starting 100 ms after stimulus onset. The authors take this finding as support for a special neuroanatomy in synesthetes and for the perceptual nature of grapheme–color synesthesia.

The differences between synesthetes and controls, however, might also be attributable to the control subjects’ ignorance of the color-related aspects of the tested graphemes. Brang, Kanai, et al. (2011), therefore, extended their previous results by testing four different control groups. Naive controls and trained controls, who learned 10 grapheme–color pairings from their matched synesthetes prior to the EEG experiment, were tested as described earlier. Another control group viewed objectively chromatic graphemes in the grapheme condition. The fourth group was also trained but were not presented with color word or patch endings, only the trained achromatic graphemes, which they were explicitly instructed to visualize to account for anticipatory effects. As in their previous study, sentences ending with a color term or patch did not produce any group differences. The N1 component was more negative in response to congruent graphemes compared to incongruent ones in synesthetes. Controls viewing chromatic graphemes also showed this trend, suggesting that this component reflects, indeed, processing of colored shapes regardless of whether the color is synesthetic or real. The congruency effect in the P2 component was observed in synesthetes only. The N400 showed a congruency effect in synesthetes and controls viewing real–colored graphemes as well as controls visualizing the anticipated grapheme. Controls trained on synesthesia-like associations also showed a trend for more negative N400 on incongruent trials. In conclusion, early components of a congruency effect in the ERP signal occurred with synesthetic color in synesthetes and real color in controls, while the later component of context sensitivity was also visible in trained controls.

Brang et al. (2010) used MEG to assess the time course of neural activity in response to synesthetic stimulation in two distinct cortical regions, V4 and the posterior temporal grapheme area (PTGA). These regions of interest (ROIs) were defined a priori by measuring the MEG responses to red squares (V4) and colored graphemes (PTGA) in the posterior temporal lobe. In the main task, achromatic graphemes and nongraphemic stimuli were presented in an upright or italic discrimination task. Both synesthetes and nonsynesthetes showed activation in response to achromatic graphemes in PTGA, while only synesthetes showed significant activation in V4. Furthermore, the V4 activation occurred only about 5 ms after the activation in PTGA, suggesting a direct connection of these two ROIs in synesthetes. The early time point suggests a response to graphemic features before the letter is identified as a whole, in line with the cross-activation theory. It would be interesting to examine possible activations in response to fake letters with similar features. The results for the nongraphemic stimuli are not presented by Brang et al. (2010). It could be argued that the PTGA, which was defined using colored graphemes, is not an exact reflection of pure grapheme processing. However, the area was defined excluding V4 to control for color-processing influences. The achromatic graphemes in the main task also elicited similar activation in this area in controls although it was slightly delayed (115–119 ms in controls, 105–109 ms in synesthetes). A comparison of the achromatic grapheme processing in synesthetes and the colored grapheme processing in controls would be interesting at this point. Still, this finding is promising in regard to the cross-activation theory. A bigger sample size (here 4 per group) with varying phenomenology in the synesthetic experience would be needed for confirmation.

### Findings Regarding Brain Structure and Function

#### Neuroanatomy

The central tenet of the cross-activation theory is that activity in V4 is induced by structural connections from grapheme-processing areas like VWFA. The first direct proof of enhanced structural connectivity in synesthetes was found by Rouw and Scholte (2007) using diffusion tensor imaging. With this technique, microstructural differences can be analyzed together with the tractography of neural fibers. Measuring the fractional anisotropy (FA) as an indicator of coherent white matter in 18 grapheme–color synesthetes plus matched controls, they found increased connectivity in the right inferior temporal cortex but also the left superior parietal and the superior frontal cortex of synesthetes. While promising, this finding could not be replicated to date (Hupé et al., 2012; Jäncke, Beeli, Eulig, & Hänggi, 2009). Whitaker et al. (2014) found overall decreased FA and increased perpendicular diffusivity in synesthetes compared to nonsynesthetes. This finding was ascribed to more crossing pathways in the brain of synesthetes. Within synesthetes, the microstructural measurements correlated with scores on the Vividness of Visual Imagery Questionnaire (Marks, 1973), as synesthetes with very vivid imagery showed the lowest FA values (Whitaker et al., 2014). Note, however, that their study was criticized for an insufficient measuring technique (six directions, 1.5 Tesla, cluster extent statistics; see also Hupé & Dojat, 2015).

Another approach to study structural brain anatomy is measuring gray matter volume with voxel-based morphometry (VBM). A few of the carried out studies have reported increased gray matter volume in synesthetes’ color-processing areas (Banissy et al., 2012; Jäncke et al., 2009; Weiss & Fink, 2009; see Hupé & Dojat, 2015; Janik McErlean & Banissy, 2017 for overview). Notably, there were no significant differences in cortical density between synesthetes and nonsynesthetes on a whole-brain analysis level but only in a priori defined ROIs (atlas based in Weiss & Fink, 2009 using their coordinates in Banissy et al., 2012). A study by Rouw and Scholte (2010) revealed distinct structural differences for synesthetes who experience the synesthetic color in the outside world (so-called *projectors*, increased gray matter volume in V1) and for those who experience the concurrent internally (*associators*, increased gray matter volume in hippocampus). Both types of synesthetes had increased gray matter volume in the superior parietal cortex.

#### Neurophysiology

While not stated explicitly in the cross-activation theory, it is straightforward to assume that increased structural connectivity affects the excitability of the connected cortices (Biane, Scanziani, Tuszynski, & Conner, 2015). Indeed, there is evidence for an increased excitability of the primary visual cortex in synesthetes compared to controls. Barnett et al. (2008) conducted an ERP study with Gabor patches that did not induce color experiences for any participant. Still, amplitudes of the C1 component (<90 ms) were higher for synesthetes compared to controls for a subset of stimuli with high spatial frequencies. In line with this finding, a TMS study revealed that the minimum stimulation intensity that was necessary to produce a phosphene sensation was three times lower in synesthetes compared to controls (Terhune, Tai, Cowey, Popescu, & Cohen Kadosh, 2011). The difference was specific for the visual cortex and did not occur in a motor threshold control condition. Thus, synesthetes exhibit a hyperexcitability of the visual cortex compared to controls.

In this context, it is remarkable that synesthetes and controls show differences in visual perception, beyond grapheme and inducer processing. Synesthetes who experience color concurrents also exhibited a superior performance compared to controls in a color discrimination task (Banissy et al., 2013; Banissy, Walsh, & Ward, 2009). Moreover, in a reaction time experiment with redundant visual and auditory stimuli, synesthetes showed stronger multimodal integration than controls, that is, a larger reaction time advantage relative to unimodal conditions than would be expected from processing two signals separately (Brang, Williams, & Ramachandran, 2012). These findings suggest that differences between synesthetes and controls are not confined to grapheme representations and associated colors. Rather, the synesthetic experience appears to be only one symptom of a more general difference in brain structure and function.

#### Anterior temporal lobe

While activation in visual association areas in the posterior occipito-temporal cortex has been found in some studies on synesthesia (see Rouw et al., 2011 for overview), no direct link between the ATL and synesthesia has been identified yet. The ATL lies in a methodologically difficult area of the brain to examine with MRI, as described by Chiou and Rich (2014). Lately, more research has been published regarding the ATL and its role in object knowledge in nonsynesthetes (Chiou & Lambon Ralph, 2016, 2017; Chiou, Sowman, Etchell, & Rich, 2014; Coutanche & Thompson-Schill, 2015). Hence, we are looking forward to the investigation of the ATL in the context of synesthesia.

### Findings Regarding Inducer–Concurrent Mappings

#### Mappings based on color-diagnostic words

A growing body of work indicates that the mapping of the inducers to their concurrents is not random in the synesthetic population. Rich et al. (2005) found patterns of grapheme–color pairings consistent over a large group of synesthetes. Letters that are the first letter of a color word (e.g., ‘R’ for ‘red’ or ‘Y’ for ‘yellow’) are often associated with this hue. Another pattern could be found on a more semantic level between the letters ‘D’ and brown, ‘A’ and red and ‘J’ and orange. While these letters have no phonetic link to their associated colors, semantic connections are possible, for example, the letter ‘D’ could be learned with the word ‘dog’ and dogs are often depicted with brown fur (Rich et al., 2005). The color red is typically associated with warning, as could the letter ‘A’ for attention. Another possible semantic connection could be a red apple, and ‘J’ might be linked to orange via the word ‘juice’. Furthermore, Simner et al. (2005) also compared a sample of English-speaking controls and a sample of German-speaking controls. Both groups chose similar grapheme–color pairings for colors with similar names in both languages (e.g., ‘White’ and ‘weiss’, ‘blue’ and ‘blau’) but assigned different colors to some letters depending on their native language (e.g., ‘purple’ in English was associated with ‘P’, while the corresponding German ‘lila’ was associated with ‘L’; Simner et al., 2005).

Interestingly, these patterns can also be seen in nonsynesthetes when asked about grapheme–color associations (Rich et al., 2005; Simner et al., 2005; Spector & Maurer, 2011). In the questionnaire study by Simner et al. (2005), nonsynesthetic controls stated color associations for letters. Consistent grapheme–color pairings were found for 16 letters regardless of a forced or free choice instruction. The free choice group also selected 13 grapheme–color matches that were similar to the synesthetes’ choices (16 overlapping grapheme–color pairings when comparing forced choice controls and synesthetes). However, the synesthetes used more detailed color terms than the control participants either due to a more extensive color vocabulary or because of their more vivid experience of the grapheme–color association (Simner et al., 2005). A relationship between grapheme frequency and frequency of color terms could only be found for synesthetes, while nonsynesthetes tended to pick typical colors at the beginning of the grapheme presentation session. This might reflect a different mechanism underlying the pairing of graphemes and colors in synesthetes and nonsynesthetes, even though an association is present in both groups (Simner et al., 2005).

#### Mappings based on exposition to objects

Synesthesia arises in childhood when literacy is acquired (Ward, 2013). It thus seems likely that the mappings between the inducers and the concurrents rely on coherent correspondences as experienced in childhood. Witthoft and Winawer (2006) describe a single case of a grapheme–color synesthete whose grapheme–color mappings closely resembled a set of colored refrigerator magnets that were popular during the synesthete’s childhood. In later studies that specific mapping was also found in larger samples of synesthetes, indicating that the letter–color associations reflect early learning (Witthoft & Winawer, 2013; Witthoft, Winawer, & Eagleman, 2015). It is important to note that early childhood experiences are highly individual and do not necessarily correspond to typical environmental regularities (Yon & Press, 2014). Multiple sources may influence the acquisition and modification of associations over an extended period of time, making it challenging to trace back any inducer–concurrent correspondence to a single origin (Hupé & Dojat, 2015; Simner, 2012a, 2012b; Yon & Press, 2014). For example, in a large-scale survey study, Rich et al. (2005) could not find school supplies that matched the common synesthetic mappings.

#### Trained mappings in nonsynesthetes

If grapheme–color mappings in synesthetes rely on learning, then it should be possible to establish similar mappings in nonsynesthetes. Indeed, various studies have demonstrated successful training of grapheme–color associations in neurotypicals (Bor, Rothen, Schwartzman, Clayton, & Seth, 2014; Colizoli, Murre, & Rouw, 2012, 2014; Colizoli et al., 2016; Meier & Rothen, 2009; Rothen, Schwartzman, Bor, & Seth, 2018; Rothen, Wantz, & Meier, 2011).

Meier and Rothen (2009) reported that nonsynesthetes showed a synesthetic Stroop effect after training grapheme–color associations. In an extension of this study, two training conditions were compared (Rothen et al., 2011): a nonadaptive training that consisted of a congruent or incongruent decision task with regard to the predefined associations and an adaptive task in which the right color had to be chosen among four color patches presented together with a black digit. After feedback, participants had to adjust the luminance of the congruently colored digit. Both trainings consisted of 10 sessions on consecutive days. Both training programs led to a significant enhancement of a digit–color priming effect (i.e., higher reaction time if an achromatic digit prime was congruent to the target color), indicating that trained inducers can also trigger automatic color perception (Meier & Rothen, 2009; Rothen et al., 2011). However, this effect is not entirely convincing, as the authors did not corroborate these psychophysical findings with a neuroimaging study. In the study by Brang, Kanai, et al. (2011) described in Section “Findings Regarding the Concurrent Color,” the trained control groups showed an expectancy effect in the N400 ERP component but not the differences in earlier components specific to synesthetes.

Colizoli et al. (2012, 2014) showed that also reading books with systematically colored letters led to implicit grapheme–color associations. After reading for 2 to 4 weeks, participants showed a significant congruency effect in a synesthetic Stroop task for their trained associations. These results were confirmed in a more recent study (Colizoli et al., 2016) in which also training-related neuronal activation was measured using fMRI. A localizer for VWFA and retinotopic mapping for V1 to V4 were acquired before the training. After the training, participants passively viewed trained, untrained, and colored untrained letters in a blocked design (synesthetic color localizer). All predefined areas showed higher activity for colored letters than for both achromatic sets. Trained graphemes led to more negative activation than untrained (achromatic) graphemes, especially in V1, V2, and V3. The strength of this deactivation did not correlate with the strength of the Stroop effect, though there was a slight trend. The decreased V4 activity for trained compared to untrained graphemes correlated with subjective ratings of associative color experiences. This was evaluated using a projector–associator questionnaire that assesses the characteristics of concurrent color experiences in synesthetes on a scale ranging from seeing the color in the outside world (*projectors*) to having a strong inner feeling of the grapheme–color association (*associators*; Rouw & Scholte, 2007). Participants rated their trained association as neutral (if they disagreed with all questions) or toward the associator-type. Since the training was an implicit associative learning task, it seems reasonable that participants would describe their experience in this way. The differences in these learned synesthesia-like experiences seem to be reflected in V4 activation (Colizoli et al., 2016).

There have also been studies with more extensive training. A 9-week long program consisting of various adaptive tasks induced not only a synesthetic Stroop effect but also self-reported synesthesia-like experiences for a short period of time (Bor et al., 2014). A recent study by Rothen et al. (2018) examined the consequences of thorough overtraining on self-reported ratings and behavioral as well as neuronal excitability measures. Sixty-minute training sessions on 13 letter–color associations were conducted on 5 days per week for 5 weeks. Participants stayed naive to the study goals and were only asked about color associations after all behavioral and neural data acquisition was completed. In line with the self-reported phenomenology similar to that of synesthetes (i.e., experiencing color induced by achromatic graphemes without effort), the grapheme–color associations were also evident on a behavioral level as measured by a grapheme–color consistency test (Eagleman et al., 2007, described in “Introduction” section) and a synesthetic Stroop task. To investigate the cortical changes due to training, phosphene and motor thresholds were determined using TMS as had been previously carried out with synesthetes (Terhune et al., 2011, see “Findings Regarding Brain Structure and Function” section). Indeed, the extensive training of grapheme–color associations led to a significant reduction in the phosphene threshold, indicating increased cortical excitability for colors comparable to that of genuine synesthetes. As a further manifestation of neurophysiological changes, participants showed enhanced visual evoked potentials when presented with checkerboards after the training. Again, this shows enhanced visual perceptual processing similar to that of genuine synesthetes (Barnett et al., 2008, see “Findings Regarding Brain Structure and Function” section). Extensive training of letter–symbol associations led to a congruency effect in a Stroop-like priming test with congruent or incongruent symbols but not to cortical changes or synesthesia-like phenomenology. Repeated testing in a control group without training did not provoke any of these effects.

## Conclusions

In this review, we examined two current theories of grapheme–color synesthesia. The cross-activation theory by Hubbard and Ramachandran (Ramachandran & Hubbard, 2001; Hubbard, Brang & Ramachandran, 2011) focuses on atypical perceptual processing in synesthetes, especially a direct coactivation of color-processing neurons in V4 by visual features of graphemes enabled by a structural hyperconnectivity in the brain of synesthetes. On the opposite, the conceptual-mediation model by Chiou and Rich (2014) proposes that conceptual representations of graphemes comprise enhanced color associations in synesthetes and, depending on how far the activation spreads within the semantic network (i.e., reaching high- or low-level color representations), lead to the experience of color with varying strength.

Based on studies focusing on properties of the inducer, we found more evidence for conceptual, as opposed to featural, inducer properties influencing the perception of a concurrent color. While similar features in graphemic shapes were found to be accompanied by similar concurrent colors, the finding could be explained both by coactivation (Brang, Rouw, et al., 2011) or semantic learning (Jürgens & Nikolić, 2012). Notably, the similarity of graphemes in the study by Brang, Rouw, et al. (2011) was not assessed based on physical features but on perceived resemblance which might also encompass conceptual similarities. The findings supporting that the perception of the inducer is modulated by its context and meaning are not free of methodological problems either. Morphing and rotating graphemes, as done by Bridgeman et al. (2010) and Kim et al. (2013), affect not only the identification of a letter but also its visual appearance, that is, its features. Dixon et al. (2006) conducted a well-controlled experiment with context modulation but only report results for a single case. Several experiments revealed that low levels of attention to the inducer produce low levels of interference in synesthetic Stroop tasks. However, it was not fully abolished in most cases. Thus, attention is presumably not necessary for the synesthetic experience but modulates the strength of the concurrent color experience. Most convincing are the findings of different inducers evoking the same color when they have the same meaning (Mroczko et al., 2009) and experiences of synesthesia induced by concepts. Despite some methodological shortcomings, behavioral studies using manipulations of the inducer seem to be in agreement with the idea that the conscious recognition and conceptual interpretation of the inducer are essential for the emergence of concurrent colors, as proposed by the conceptual-mediation model.

Regarding the concurrent color, neither behavioral nor fMRI data have yet provided clear evidence for or against either of the discussed theories. Concurrent colors are not identical to real colors, as one would assume if their processing is activated early on. In that case, they would induce comparable color aftereffects or pop-out effects in visual search tasks. Activity in the color-sensitive area V4 during concurrent color experiences has been shown in some but not all fMRI studies. Easy tasks like font discrimination or passive viewing of graphemes seem to promote positive results (Hubbard & Ramachandran, 2005; Sperling et al., 2006). In any case, neural activity in V4 for inducers is compatible with both models (bottom-up in the cross-activation theory, but top-down in the conceptual-mediation model). Analyses of the time course of inducer processing by means of EEG and MEG provide strong evidence for atypical perceptual processing in synesthetes. Especially, the reported early coactivation of V4 measuring MEG source activity during inducer processing is in line with the predictions of the cross-activation theory (Brang et al., 2010). It can be objected that MEG source reconstructions lack the spatial resolution that would be necessary to distinguish between activity in the neighboring VWFA and V4 sites (Krishnaswamy et al., 2017). However, together with the results of the above reviewed fMRI studies, this finding favors a perceptual explanation for synesthesia. In sum, behavioral findings suggest that synesthetic colors are different from real colors, but selected fMRI studies have revealed increased BOLD responses in color-sensitive area V4 during inducer processing, likely due to early coactivation as revealed by EEG/MEG studies.

The examination of structural differences between synesthetes and nonsynesthetes revealed increased FA in the right inferior temporal cortex in one study (Rouw & Scholte, 2007) and decreased FA in the whole brain in another study (Whitaker et al., 2014). VBM revealed increased gray matter volume in color-sensitive areas in synesthetes (Banissy et al., 2012; Jäncke et al., 2009; Weiss & Fink, 2009). However, not all studies researching structural differences in synesthetes are in agreement with these findings (Hupé & Dojat, 2015). The probably small differences might be hard to detect with current methods in DWI and VBM. Hence, further research is needed to replicate these promising findings of atypical brain anatomy. Synesthetes also show structural differences in other areas, such as increased FA values in the parietal cortex (Rouw & Scholte, 2007) and increased gray matter volume in the hippocampus (Rouw & Scholte, 2010). The former is in line with the revised cross-activation theory, which suggests enhanced binding in the parietal cortex as one supporting mechanism in synesthesia. Increased hippocampus size might hint at an increased memory capacity also in line with the conceptual-mediation model. Besides structural differences, hyperexcitability of the visual cortex in response to nonsynesthetic stimuli has been found using EEG (Barnett et al., 2008) and TMS (Terhune et al., 2011). Together with behavioral evidence of enhanced visual perception (Banissy et al., 2009) and multisensory integration (Brang et al., 2012) in synesthetes, this hints at a more general altered brain function in this population. Enhanced microstructure and excitability in synesthetes overall support the cross-activation theory. The involvement of the ATL has not been sufficiently investigated yet.

Examination of both synesthetes’ and nonsynesthetes’ grapheme–color pairings shows that the mapping of colors to inducers is not random but builds on previous experiences with color-diagnostic words or objects. Various semantic associations between colors and their inducing graphemes have been identified (Rich et al., 2005; Simner et al., 2005). Moreover, nonsynesthetes seem to exhibit similar grapheme–color associations even though these do not lead to a conscious synesthetic experience (Rich et al., 2005; Spector & Maurer, 2011). Training programs have been shown to effectively induce synesthesia-like behavior, for example, the congruency effect, and even changes in neuronal activity in nonsynesthetes (Bor et al., 2014; Colizoli et al., 2012, 2014, 2016; Meier & Rothen, 2009; Rothen et al., 2011, 2018). However, the long-term training effects need to be evaluated further, as self-report in one study indicated that these were not permanent (Rothen et al., 2011). Since synesthetes experience their concurrents automatically since childhood, they are essentially rehearsing their color associations in a lifelong training. Hence, it is plausible that even training sessions on several consecutive days may be insufficient to mimic synesthesia to its full extent. An intensive training regime over several weeks also induced cortical changes (Rothen et al., 2018), suggesting that a large amount of exposure to and active engagement with the grapheme–color associations could account for the neuronal differences seen in synesthetes. This is in line with the conceptual-mediation model, as it states that the strength of the synesthetic experience depends on the strength of the associations in the memory network.

Overall, the following picture emerges: The prevailing findings regarding the properties of the inducer and the inducer–concurrent mappings broadly support the conceptual-mediation model. On the other hand, the available findings in relation to concurrent colors and brain structure support the cross-activation model. Given that the empirical evidence does not favor one or the other theory, but rather specific predictions thereof, we will try to offer an integrative view on the data.

There seem to be individuals whose neural endowment makes them prone to synesthetic experiences. They are characterized by highly excitable and hyperconnected brains. From a computational point of view, high connectivity allows for high degrees of freedom in cross-modal representations (Bavelier & Neville, 2002). Moreover, a high excitability promotes high learning rates and thus functional plasticity (Holtmaat & Caroni, 2016). These neural characteristics would also promote a cross-modal mixing of color and shape information, as in grapheme–color synesthesia. Although synesthetes’ color and letter associations can already be observed in early childhood, at the age of 6 (Simner et al., 2009), the consistency of the letter–color mappings increases with age. This supports the idea that grapheme–color synesthesia results from an interaction of neurostructural and environmental (i.e., learning) factors. While structural connections and direct cross-activation might enable the initial link between inducers and concurrents, these associations are stored and reinforced in a conceptual knowledge network. Consequently, the activation of the concept alone would suffice for a concurrent color experience. We have argued in a previous work that color coactivation may then become increasingly automatic due to long-term potentiation (Volberg et al., 2013). As a result, an inducer would evoke activity in color-sensitive areas early during processing.

Since nonsynesthetes also exhibit color associations for graphemes (Spector & Maurer, 2011; Simner et al., 2005), it can be argued that a color-link in the semantic network is not exclusive to synesthetes. Indeed, the synesthetic condition has been considered as an extreme along a continuum of cross-sensory associations of different intensities (Simner, 2012a, 2012b). It is reasonable to assume that the conceptual knowledge of graphemes is alike in synesthetes and nonsynesthetes with a common language. Conceptual knowledge on letter colors would be stored in the same network as previous perceptual color experiences with that letter. With age, the associated color might then converge more and more to the canonical hue found in the population.

To date, both the cross-activation and conceptual-mediation theories have their eligibility. It remains to be investigated which of the proposed mechanisms are crucial for the synesthetic experience. Research on grapheme–color synesthesia is deeply rooted in visual neuroscience and theories of multisensory perception (Brang et al., 2010; Brang, Kanai, et al., 2011; Grossenbacher & Lovelace, 2001) and less in memory research or color vision. In the last section, we want to consider how the idea of synesthesia as a form of object knowledge could be investigated applying current paradigms used in memory color research.

## Future Directions

In a recent study with synesthetes and controls, Volberg, Chockley, and Greenlee (2017) found a pattern of EEG theta activity specific to synesthetes during the processing of inducing and noninducing graphemes. Since the graphemes were presented as distractors within an orientation judgement paradigm and so were fully irrelevant to the task, this suggests that graphemes that would later induce colors are automatically selected and processed in synesthetes. The automated processing of graphemes is possibly an important clue to understand grapheme–color synesthesia. The enhanced encoding was attributed to increased grapheme expertise (Volberg et al., 2017). Expertise implies an increased scrutiny and preference for the inducers. Indeed, synesthetes show significantly more pronounced interest in literature and language than nonsynesthetes (Rich et al., 2005). Furthermore, synesthetes exhibit a distinct cognitive style favoring verbal and vivid imagery (Meier & Rothen, 2013). This might be linked to synesthetes’ higher scores than controls’ in verbal originality, vocabulary, and usage of mental imagery (Chun & Hupé, 2016). Of course, it remains to be determined whether these traits are a consequence of the synesthetic experience. However, in light of the findings reviewed earlier, it seems fair to assume that these traits make synesthetes more prone to building extensive association networks involving graphemes and promote the development of synesthesia.

If synesthetic mappings rely on abstract representations in an association network, the colors should be experienced whenever a grapheme triggers the retrieval of the color from memory. To investigate the relationship between synesthesia and memory for colors, Arnold et al. (2012) compared the matching precision of synesthetic colors with real and memorized colors in synesthetes who only experience color when seeing (but not when hearing) a grapheme. Both synesthetes and control participants performed better during real color perception, than during recollection, in line with findings for memorized color in nonsynesthetes (Knill & Richards, 1996). Moreover, synesthetes were just as precise when recalling their synesthetic colors as during the concurrent perception. This suggests that synesthetic color experiences may involve a retrieval of color information from memory, triggered by the grapheme as a retrieval cue.

Associations between objects and colors are not limited to grapheme processing in synesthetes but occur commonly in the normal population. The typical color of an object that is learned through experience is referred to as a memory color (Bartleson, 1960; Hering, 1920; Olkkonen, Hansen, & Gegenfurtner, 2008; Witzel, Valkova, Hansen, & Gegenfurtner, 2011). These memory colors have an impact on our real-world perception. Achromatic pictures of objects are perceived as slightly colored in the objects typical colors, as shown by Hansen et al. (2006), Olkkonen et al. (2008), and Witzel (2016). This effect of object knowledge on color perception is called the memory color effect. Hansen et al. (2006) implemented a method to determine how much tint of the opponent color an achromatic image needs to cancel out the object’s memory color. In an achromatic color adjustment task, participants adjusted the color of natural objects (fruits) to neutral gray. However, participants overcompensated by adjusting the color of the object to be slightly tinted in the opponent color instead of neutral. It appears that a slight opponent color hue is necessary to cancel out the memory color for this object in order for it to appear gray. For example, an image of a banana was only perceived to be gray when it was actually adjusted to have a slightly blue color. The memory color effect is still evident under different illumination conditions (Olkkonen et al., 2008). Witzel et al. (2011) further investigated the memory color effect for artificial objects. They first measured the color diagnosticity for each object (i.e., how much an object is linked to a typical color) and then conducted an achromatic color-adjustment task. In line with findings for natural objects, knowledge about the typical color influenced the color appearance (Witzel et al., 2011). The memory color effect depends on the degree of familiarity with an object. It occurs only for highly familiar objects, for example, brand logos with high compared to low number of stores (Kimura et al., 2013). Taking into consideration these current findings on memory colors, we deem further research on its role in synesthesia necessary. The fact that knowledge about the typical color of an object modulates how the object is perceived, especially in highly familiar objects (like graphemes are for synesthetes), has not yet been appropriately examined in synesthesia research.

Acting on the assumption that the extent of trait expression exhibited by synesthetes and nonsynesthetes form a continuum (Simner, 2012a, 2012b), the strength of the memory color effect could provide a measure of the strength of the inducer–concurrent association with a cutoff differentiating between synesthetes and nonsynesthetes. The achromatic adjustment task (Hansen et al., 2006) is an elaborate paradigm. As an alternative, Witzel (2016) proposed a forced choice task to show the memory color effect in a large sample group. He conducted a series of online surveys which included displays of a pair of bananas or a pair of disks, one of them in the neutral background gray and the other in a slightly bluish tint. Participants had to decide which one of the pair was purely gray. Consistent with his hypothesis, participants picked the bluish banana significantly more often than the neutral gray banana. This effect was not significant for the disks.

We suggest that the display task by Witzel (2016) might be adapted to examine synesthetic colors by replacing the bananas with inducers and the disks with noninducing control stimuli. The coloring of the stimuli would be purely gray or with a slight tint in the opponent color of the individual concurrent color for each tested inducer. According to the memory color effect, synesthetes should pick the colored stimuli over the natural gray ones on inducer trials. Such experiments could provide support for the idea that grapheme–color synesthesia is related to the formation of memory colors.
